# Challenges and opportunities in coordinating investments across countries and channels to expand access to a new contraceptive method

**DOI:** 10.1186/s12905-025-03949-z

**Published:** 2025-09-03

**Authors:** Kaleigh MacDaniels

**Affiliations:** The Bill and Melinda Gates Foundation, 500 5th Ave N, Seattle, WA 98109 USA

**Keywords:** Self-care, Contraceptive self-care, DMPA-SC, Self-injection, Contraceptive self-injection

## Introduction

Offering contraceptive choice is critical to ensuring that those who want to use contraception can find the options that best meet their needs and preferences throughout their life course. Subcutaneous depot medroxyprogesterone acetate (DMPA-SC), an all-in-one, lower-dose injectable contraceptive, represents one way to expand choice because administration does not require a highly trained healthcare provider. Instead, DMPA-SC is easy enough to administer that any trained person can do it, including community health workers—known as provider administered—and women themselves—known as self-injection. Self-injection, jointly with other self-care methods, can help overcome traditional barriers to access and use of contraception, resulting in women and girls having increased control of their reproductive health, which can lead to reduced disparities and improved health, human rights, and social outcomes.

To support access to self-injection, the Bill & Melinda Gates Foundation and the Children’s Investment Fund Foundation (CIFF) funded partners to embark on a series of coordinated implementation projects in Kenya, Malawi, Nigeria, Pakistan and Uganda from 2019 to 2024. These projects were designed to understand how to successfully introduce and scale-up access to self-injection in a variety of geographies and channels. Today, thanks in part to these partners and projects, which are represented in this special supplement, millions of people have access to self-injection should they want it. Self-injection of DMPA-SC is available in at least 19 countries and there have been more than 1.2 million visits for women seeking self-injection. In the 14 countries with DMPA-SC service statistics disaggregated by mode of administration, around 22% of DMPA-SC visits are for self-injection, with the remaining 78% for provider-administered injections [1] Despite this, there is still more work to be done in terms of how donors like the Gates Foundation make coordinated investments, especially if comparability is needed. Some of the challenges include how donors and the broader community measure and protect reproductive autonomy and free choice in the context of new method introduction; and how policies, guidelines and practices show trust and empower to women and girls.

## Background

After decades of device and product development by organizations such as PATH, Becton, Dickenson and Company (BD), the United States Agency for International Development (USAID) and Pfizer, the Gates Foundation started funding supply security and delivery support for DMPA-SC in 2008. This was in recognition of the potential for the method to expand choice for women and girls seeking injectable contraception. Initially, the Foundation’s downstream DMPA-SC strategy focused on supporting our funded partners to execute an expanded agenda of global- and field-based research. The work was focused on generating evidence on DMPA-SC’s benefits and the method’s value proposition. While, at the time, the method’s core value was thought to be increasing the number and type of providers delivering injectable contraceptives in low-and middle-income countries, the Gates Foundation and its partners, were also eager to understand if self-injection could be acceptable and feasible from the perspective of potential users, providers and health systems.

PATH, with support from the Gates Foundation, conducted a qualitative study on self-injection in Ethiopia in 2012. While the study did not involve actual use of self-injection due to regulatory requirements, women in Ethiopia were able to test the method on a model and found it easy to use, and they valued the time and expense that could be saved [2]. With funding from USAID, FHI 360 conducted an observational study in Senegal and Uganda from 2012 to 2013. 45% of women in Uganda and 22% of women in Senegal said that they were moderately or very willing to self-inject DMPA-SC after receiving DMPA-SC from a provider [3].

After the initial research on self-injection, Senegal and Uganda secured regulatory waivers so that clients could self-inject, and the respective Ministries of Health worked with partners to conduct operational feasibility, effectiveness, and cost-effectiveness studies. The study results indicated that self-injection is both feasible and acceptable to women: Around 90% of the participants in Senegal and Uganda were deemed proficient in self-injection three months post-training, and over 93% of participants in Senegal and Uganda said they wanted to continue to self-inject [4, 5]. Subsequent studies in the same two countries found that continuation rates for women self-injecting DMPA-SC were significantly higher than for women who received intramuscular (IM) DMPA from a provider, an indication that self-injection may allow women to circumvent barriers to accessing contraception and more consistently use contraception when they desire pregnancy prevention [6, 7].

## Self-injection implementation projects

In 2019, with the growing evidence on the value of self-injection in hand, Ministries of Health looked to approve and implement self-injection policies. With this shift in focus within countries, the Gates Foundation evolved our strategy from a primary focus on supporting provider-administered DMPA-SC in the public sector to include a strong focus on self-injection in a variety of sectors and channels. Until this point, use of self-injection was low, and, while it was known that some women and girls could benefit from access to self-injection, there was a limited understanding of how best to support that access. As such, the Gates Foundation developed a delivery strategy centered around a coordinated set of focused implementation projects intended to test different delivery channels in different contexts. This work was designed to understand (1) how self-injection could best be introduced into a specific market and/or channel to expand the contraceptive options available to women and girls, considering health system characteristics and women and girls’ needs; and (2) the demand for self-injection when DMPA-SC was offered alongside a range of other methods.

To select the markets and channels for the implementation projects, the Gates Foundation looked at indicators such as modern contraceptive prevalence rate (mCPR), method mix, most recent source of method among injectable users, discontinuation rates among injectable users, and the status of DMPA-SC introduction in different countries. We selected diverse markets and channels to understand which markets and channels were better suited to meet the needs of women and girls through accessing DMPA-SC and self-injection, and to develop global best practices related to the introduction and scale-up of self-injection. A series of global and country-specific learning questions drove the design of each implementation project. Ultimately, the Foundation chose the markets and channels in Table 1.

**Table 1:** Self-injection implementation projects

Geography	DMPA-SC first introduced	Channels(s) to be tested/scaled	Objective	Archetype
Kenya	2017	Retail private sector	Testing the role of the retail private sector for self-injection in a country with high mCPR and injectables use overall but low DMPA-SC use to understand if offering self-injection in the retail private sector could meet the needs of existing and potential users of injectable contraception	Higher mCPR, higher injectables use, lower DMPA-SC use
Malawi	2018	Public sector	Demonstrating scale-up of self-injection in a country that started with self-injection and provider-administration from the beginning (vs. starting with provider-administered DMPA-SC only and then adding self-injection)	Higher mCPR, higher injectables use, higher DMPA-SC use
Nigeria	2014	Total market approach	Demonstrating scale-up of self-injection in the public sector and testing self-injection in the private sector to understand if DMPA-SC and self-injection could lead to increases in localized mCPR	Lower mCPR, higher injectables use, higher DMPA-SC use
Pakistan (Punjab Province)	2018	Public sector and private midwives	Testing the introduction of self-injection and provider-administration concurrently in a country with low injectables use overall to understand if DMPA-SC and self-injection could lead to increases in localized mCPR	Lower mCPR, lower injectables use, lower DMPA-SC use
Uganda	2014	Public sector	Demonstrating scale-up of self-injection in a country that started with provider-administered DMPA-SC only	Higher mCPR, higher injectables use, higher DMPA-SC use

## Self-injection learning exchange

It was important for the Gates Foundation to approach the self-injection implementation projects as a coordinated portfolio rather than one-off investments because we wanted to (1) be able to look across investments to compare results and impact, which was especially important to guide our strategy and investments internally; (2) enable our partners to share learnings with one another; and (3) work with our partners to identify best practices. Toward that end, we engaged the University of California San Francisco (UCSF) to help us achieve these goals. UCSF developed the Self-Injection Learning Exchange, a collaborative group focused on aggregating and synthesizing partner learnings, sharing resources, and facilitating discussions of emergent findings among our partners. In addition to our own partners, the Self-injection Learning Exchange includes partners funded by CIFF.

## Learnings

### Comparing results and impact

While we wanted to compare results and impact across geographies, this was difficult for several reasons. First, the unique dynamics of each health system and the funding availability meant that the different partners identified diverse measurement approaches that made it difficult to identify a set of standardized indicators across all the implementation projects. For example, several of our partners prioritized working within existing data collection systems and therefore relied on health management information systems (HMIS) and were therefore limited by what was tracked there. Other partners worked with the private sector, which had its own challenges regarding indicators and data access.

Second, each project faced externalities that impacted the results. For example, several of the geographies faced stockouts of DMPA-SC that hampered access and, ultimately, impact. Policies also played an important role. For example, Malawi has a policy that a woman who is trained on self-injection can take home three units of DMPA-SC after the training, while Nigeria used to require a woman to come back for a second visit before she was allowed to take units home (this policy has since changed to allow women to take home three units after their initial training). In addition, cultural and societal norms varied widely across geographies.

The lack of consistent indicators and the presence of different externalities meant that it was difficult to establish comparative effectiveness across geographies. Instead, the Self-injection Learning Exchange relied more on qualitative assessments of impact when making comparisons. While the Self-injection Learning Exchange captured some quantitative data on priority indicators across partners and countries, given the heterogeneity of projects and country contexts, the comparisons were not truly apples to apples. In the future, if definitive comparative effectiveness is needed, we will need large-scale research investments that are designed foremost for comparability.

### The value of the self-injection learning exchange

Despite cultural and policy differences across geographies, and the measurement challenges to comparing results across investments, sharing learnings through the Self-injection Learning Exchange was valuable in several ways. First, coordinating efforts across the geographies and projects helped to address some of the challenges like supply availability, a reoccurring and persistent issue across settings. Partners were able to share strategies and interventions that worked with one another, and they were able to work together to advocate for additional supply chain-related resources within geographies. Second, the partners were able to collaborate to innovate on the measurement of informed choice in the context of method introduction.

The Self-injection Learning Exchange supported coordination through quarterly meetings with all the implementation project partners and the donors (CIFF and Gates Foundation). These deep dive meetings were used to share lessons learned and to discuss challenges engendered by group problem-solving. These meetings and the Self-injection Learning Exchange, more broadly, created an environment that was not competitive but one of learning and sharing. Indeed, this special supplement is one example of how the partners worked together to comprehensively answer some of the most challenging learning questions across geographies around how to make DMPA-SC self-injection more accessible to women should they want to choose this method and mode of administration.

### Ensuring reproductive autonomy and free choice

As evidenced by Senderowicz et al. (2022), vertical and method-specific health programs that are evaluated on a narrow set of programmatic outcomes can end up limiting choice and autonomy. As such, we struggled to define success for this work [8]. We needed outcomes and indicators that could show accountability internally for our product-specific funding, but we were highly cognizant that any internal goals could lead to bias and/or directive counseling in the field. Ultimately, we set a goal for seeing an overall increase in contraceptive use because the work was meant to first and foremost support full, informed and free choice of any method, and because much of the work was focused on systems strengthening that would benefit the whole system and any method. We also set a goal of seeing increases in the proportion of DMPA-SC that is self-injected, as we felt this was an important indicator of increasing access to self-injection. The Gates Foundation did not set any targets to measure the increases, and worked with our partners to ensure that the choice to have DMPA-SC administered by a provider was just as important as the choice to self-inject for those women who chose DMPA-SC—this was all in the context of full, informed and free choice of any contraceptive method. As the Women’s Health Innovations team at the Gates Foundation, we continue to weigh the benefits and challenges of different measures for our product introduction work, especially measures related to the introduction of contraceptives. We are consistently seeking ways to engage our partners and the contraception research community to identify measures that uphold reproductive autonomy and free choice in the context of product introduction.

## Looking forward

This special supplement brings together some of the latest research on DMPA-SC and self-injection from a series of coordinated implementation projects in Kenya, Malawi, Nigeria, Pakistan and Uganda. While partners were able to distill some general best practices for introducing and scaling self-injection, which are available on FPOptions.org, this effort also reinforced that introducing a new method is not linear within geographies and is not the same across geographies. There are unique cultural norms and policy guidelines in each country. Contraceptive method introduction and scale requires a deep understanding of both the women and girls who may want or benefit from the method, and the health system in which the method is being introduced. Therefore, there is still effort needed at national and sub-national levels to understand how self-injection and other new and underutilized methods can fit within health and social systems. For example, when considering the private sector, what level of data is needed and how is it collected in a way that protects the privacy and discreetness that can come with seeking contraception through less formal ways (e.g. through pharmacies)? Also, how can donors, governments and partners work together to proactively plan for new and underutilized method introduction, and identify more effective and efficient paths to method institutionalization within health systems?

In addition, there is still more work to do to understand how best to measure and ensure reproductive autonomy and free choice when introducing new methods like DMPA-SC. As the Women’s Health Innovations team at the Gates Foundation, we continue to grapple with identifying the appropriate outcomes for our work on DMPA-SC and contraceptive product introduction more broadly. How should we best measure the impact of our investments in a way that does not lead to bias and protects reproductive autonomy and free choice, while still providing us with the required data to account for our product-specific funding?

Despite what we don’t know, what we do know—as evidenced through the papers in this supplement, additional research and real-world data—is that self-injection of DMPA-SC is safe, feasible, and acceptable. Unfortunately, we continue to see prohibitive policies and guidelines that limit access to self-injection. For example, some countries still require women to come back to a health provider for multiple trainings before they are given units to take home—this is even if they want to self-inject and have demonstrated that they can correctly self-inject. There can be various reasons for this; one that stands out is a distrust in women’s ability to care for themselves. We see it in the United States and in many other countries with policies that restrict access to care, and we see it with policies and guidelines—like those related to self-injection —that strip the trust and power away from women and girls. If we are to see the benefits that can come with self-injection and self-care more broadly, we need to better understand women and girls’ needs, place trust in their ability to care for themselves and give them the power to do so. We can do this by listening to women and girls and doing a better job bringing along key stakeholders, such as providers and key opinion leaders. My hope for the implementation projects and the research represented in this special supplement is that they show the benefit that comes with collaboration across projects, partners and donors and, more importantly, they show the benefit that can be gained when policies, guidelines, and practices give power and trust to women and girls.

## Data Availability

All papers and reports cited in this opinion piece are publicly accessible and can be accessed in cited sources within the text.
